# Supplementary material for: Incidence and Transition of Acute Kidney Injury, Acute Kidney Disease to Chronic Kidney Disease after Acute Type A Aortic Dissection Surgery
Source: J Clin Med. 2021 Oct 18;10(20):4769. doi: 10.3390/jcm10204769 (PMC8540632; doi:10.3390/jcm10204769)
Supplement: Supplementary file 1 [file jcm-10-04769-s001.zip › jcm-1361451-supplementary.pdf]

Supplementary Table S1. Diagnoses used to define the cohorts, comorbidities, and outcomes

Disease	ICD-9 CM disease code	ICD-10 CM disease code
**Comorbidities**		
Marfan syndrome	759.82	Q87.4
Diabetes mellitus	250	E08-E13
Hypertension	401 – 405	I10-I15, N262
Dyslipidemia		
Chronic kidney disease	580 – 589, 403 – 404, 016.0, 095.4, 236.9 250.4, 274.1, 442.1, 447.3, 440.1, 572.4, 642.1, 646.2 753.1, 283.11, 403.01, 404.02, 446.21	A1811, D593, E102, E112, E132, I12, I13, K767, M103, M310, N00, N01, N02, N03, N04, N05, N06, N07, N08, N14, N150, N158, N159, N16, N171, N172, N18, N19, N200, N25, N261, N269, N27, Q61
Old stroke	430 – 437	I60-I62, I66, I65.1, I65.0, I65.8, I65.9, I63.6, I63.8, I63.9, G45.0, G45.8, G45.1, G45.2, G46.0, G46.1, G46.2, G45.9, G45.4, G46.3, G46.4, G46.5, G46.6, G46.7, G46.8, I67.0, I67.1, I67.2, I67.4, I67.5, I67.6, I67.7, I67.9, I68.0, I68.2, I68.8
Chronic obstructive pulmonary disease	491, 492, 496	J41-J44
Coronary artery disease	410 – 414	I20-I24
Atrial fibrillation	427.3	I48
Peripheral arterial disease	440, 441, 443, 444.0, 444.8, 447.8, 447.9, 093.0, 437.3, 444.22, 447.1, 557.1, 557.9, V434	I70, I71, I73, I75, I771, I790, I791, I792, I773, I779, I798, K551, K558, K559, Z958, Z959, I743, I744, I745, I748, I740, I7789
**Outcomes**		
Respiratory failure	518	J96

Ischemic stroke	433 – 437	I66, I65.1, I65.0, I65.8, I65.9, I63.6, I63.8, I63.9, G45.0, G45.8, G45.1, G45.2, G46.0, G46.1, G46.2, G45.9, G45.4, G46.3, G46.4, G46.5, G46.6, G46.7, G46.8, I67.0, I67.1, I67.2, I67.4, I67.5, I67.6, I67.7, I67.9, I68.0, I68.2, I68.8

Supplementary Table S2. Baseline and clinical characteristics of patients according to AKI or AKD

Variables	Valid *N*	Total (*n* = 696)	AKI (*n* = 376)	Non-AKI (*n* = 320)	*P* value	AKD (*n* = 169)	Non-AKD (*n* = 527)	*P* value
AKI stage	696				--			<0.001
0		320 (46.0)	0 (0.0)	320 (100.0)		34 (20.1)	286 (54.3)	
1		170 (24.4)	170 (45.2)	0 (0.0)		31 (18.3)	139 (26.4)	
2		88 (12.6)	88 (23.4)	0 (0.0)		29 (17.2)	59 (11.2)	
3		118 (17.0)	118 (31.4)	0 (0.0)		75 (44.4)	43 (8.2)	
Demographics								
Age, years	696	57.6±13.7	57.4±13.4	57.7±14.0	0.774	57.5±13.3	57.6±13.8	0.924
Male gender	696	472 (67.8)	268 (71.3)	204 (63.8)	0.034	116 (68.6)	356 (67.6)	0.792
Body weight, kg	691	73.3±17.0	75.8±18.6	70.4±14.5	<0.001	75.0±18.2	72.8±16.6	0.148
Height, cm	669	164.5±14.6	164.5±15.3	164.5±13.8	0.990	163.1±17.3	164.9±13.6	0.155
BMI, kg/m^2	669	25.9 [23.3, 29.4]	27.1 [24.6, 29.7]	25.2 [23.0, 28.4]	<0.001	27.6 [24.7, 32.2]	25.5 [23.2, 28.5]	0.003
Smoking	696	272 (39.1)	146 (38.8)	126 (39.4)	0.883	63 (37.3)	209 (39.7)	0.581
Comorbidities								
Marfan syndrome	696	26 (3.7)	11 (2.9)	15 (4.7)	0.222	5 (3.0)	21 (4.0)	0.540
Diabetes mellitus	696	54 (7.8)	27 (7.2)	27 (8.4)	0.537	13 (7.7)	41 (7.8)	0.970
Hypertension	696	500 (71.8)	264 (70.2)	236 (73.8)	0.301	108 (63.9)	392 (74.4)	0.008
Dyslipidemia	696	58 (8.3)	34 (9.0)	24 (7.5)	0.463	20 (11.8)	38 (7.2)	0.058
Chronic kidney disease	696	76 (10.9)	58 (15.4)	18 (5.6)	<0.001	36 (21.3)	40 (7.6)	<0.001
Old stroke	696	23 (3.3)	13 (3.5)	10 (3.1)	0.807	4 (2.4)	19 (3.6)	0.433
Chronic obstructive pulmonary disease	696	29 (4.2)	15 (4.0)	14 (4.4)	0.800	9 (5.3)	20 (3.8)	0.386

Variables	Valid *N*	Total (*n* = 696)	AKI (*n* = 376)	Non-AKI (*n* = 320)	*P* value	AKD (*n* = 169)	Non-AKD (*n* = 527)	*P* value
Coronary artery disease	696	60 (8.6)	31 (8.2)	29 (9.1)	0.702	10 (5.9)	50 (9.5)	0.150
Atrial fibrillation	696	49 (7.0)	20 (5.3)	29 (9.1)	0.054	7 (4.1)	42 (8.0)	0.091
Peripheral arterial disease	696	98 (14.1)	48 (12.8)	50 (15.6)	0.280	24 (14.2)	74 (14.0)	0.959
Pre-OP vital signs								
Mean arterial pressure, mmHg	695	89.4±19.0	89.4±20.6	89.4±17.0	0.977	89.7±21.3	89.3±18.2	0.817
Heart rate, beats/min	695	83.9±18.2	84.4±19.1	83.3±17.1	0.410	84.4±19.0	83.8±18.0	0.712
Pre-OP lab data								
Creatinine, mg/dL	696	1.29±1.00	1.44±1.30	1.12±0.40	<0.001	1.58±1.59	1.20±0.70	<0.001
BUN, mg/dL	511	18.5±8.1	19.5±9.2	17.3±6.5	0.003	20.5±9.8	17.8±7.3	0.001
WBC, 10^3/uL	691	13.0±4.6	13.3±4.6	12.6±4.6	0.033	12.5±4.5	13.1±4.6	0.118
Hemoglobin, g/dL	691	13.5±2.0	13.6±2.0	13.3±1.9	0.034	13.3±2.1	13.5±1.9	0.161
Platelet, 10^3/uL	691	179.6±59.4	172.7±52.1	187.7±66.1	0.001	174.3±53.4	181.2±61.1	0.192
Sodium (Na), mEq/L	682	139.1±3.4	139.4±3.6	138.8±3.2	0.023	139.0±3.8	139.2±3.3	0.682
Potassium (K), mEq/L	682	3.7±0.5	3.7±0.6	3.7±0.5	0.949	3.7±0.6	3.7±0.5	0.978
Surgical details								
Bypass time, min	579	253.5±69.5	263.0±73.0	241.9±63.2	<0.001	268.7±89.5	248.5±60.9	0.003
Clamp time, min	576	162.0±53.1	168.2±55.1	154.3±49.7	0.002	170.5±64.9	159.2±48.5	0.027
Arrest time, min	571	50.1±24.2	52.1±26.2	47.6±21.2	0.025	51.3±27.4	49.7±23.0	0.490
HTK, cc	260	2175.4±521.0	2180.6±517.9	2170.6±525.7	0.877	2239.7±625.7	2156.9±487.0	0.287
Brain protection	696				0.441			0.499
Antegrade		374 (53.7)	197 (52.4)	177 (55.3)		87 (51.5)	287 (54.5)	
Retrograde		322 (46.3)	179 (47.6)	143 (44.7)		82 (48.5)	240 (45.5)	
Cerebral perfusion time, min	330	51.4±21.3	53.7±23.3	48.8±18.6	0.038	50.9±23.3	51.6±20.8	0.812

Variables	Valid *N*	Total (*n* = 696)	AKI (*n* = 376)	Non-AKI (*n* = 320)	*P* value	AKD (*n* = 169)	Non-AKD (*n* = 527)	*P* value
OR shock or tamponade	696	87 (12.5)	54 (14.4)	33 (10.3)	0.107	19 (11.2)	68 (12.9)	0.570
Extension for type A surgery								
Aortic arch replacement	696	239 (34.3)	140 (37.2)	99 (30.9)	0.081	64 (37.9)	175 (33.2)	0.267
Aortic root replacement (Bentall operation)	696	54 (7.8)	24 (6.4)	30 (9.4)	0.141	14 (8.3)	40 (7.6)	0.769
Elephant trunk	696	20 (2.9)	8 (2.1)	12 (3.8)	0.202	5 (3.0)	15 (2.8)	0.939
Ascending aorta replacement only	696	394 (56.6)	210 (55.9)	184 (57.5)	0.662	89 (52.7)	305 (57.9)	0.234
Post-OP status								
SOFA score	222	10.5±2.0	10.7±2.0	10.1±1.9	0.023	11.0±2.2	10.3±1.9	0.033
SOFA score, excluding total bilirubin	476	9.8±1.9	10.1±1.9	9.5±1.8	<0.001	10.4±2.2	9.6±1.7	<0.001
Post-OP lab								
AST	493	77.0 [51.0, 142.0]	84.0 [52.0, 172.0]	70.0 [49.0, 106.0]	0.002	102.0 [54.0, 196.5]	72.0 [50.0, 121.0]	0.001
ALT	421	46.0 [25.0, 98.0]	55.0 [24.0, 119.0]	37.0 [25.0, 74.0]	0.027	53.0 [25.0, 125.0]	44.0 [24.0, 90.5]	0.169
Hemoglobin, g/dL	696	10.9±1.6	10.7±1.7	11.1±1.5	<0.001	10.7±1.6	10.9±1.6	0.138
Platelet, 10^3/uL	696	136.1±43.4	131.3±40.2	141.7±46.4	0.002	129.7±39.9	138.2±44.3	0.027
Hospital stay	696	22.0 [15.0, 34.0]	26.0 [17.0, 43.0]	19.0 [14.0, 27.0]	<0.001	33.0 [21.0, 60.0]	20.0 [14.0, 30.0]	<0.001
Follow-up years	696	4.4±3.4	4.7±3.6	4.0±3.0	0.004	4.3±3.7	4.4±3.3	0.766

Abbreviations: AKI, acute kidney injury; AKD, acute kidney disease; BMI, body mass index; OP, operation; BUN, blood urea nitrogen; WBC, white blood cells; OR, operation room; SOFA, Sequential Organ Failure Assessment; AST, aspartate aminotransferase; ALT, alanine aminotransferase.

Supplementary Table S3. The association of AKD with the risk of recurrent AKI and newly diagnosed CKD in the presence of ESRD as the competing risk

**Late outcomes (>90days)**	**AKD (n = 169)**	**Non-AKD (n = 527)**	**Unadjusted analysis**		**Multivariable analysis†**	
			**SHR (95% CI) for AKD**	**P value**	**SHR (95% CI) for AKD**	**P value**
Recurrent AKI	35 (20.7)	77 (14.6)	1.56 (1.04 - 2.33)	0.031	1.24 (0.77 - 2.00)	0.386
Newly-diagnosed CKD	93 (55.0)	201 (38.1)	1.94 (1.48 - 2.53)	<0.001	2.86 (2.10 - 3.89)	<0.001

Abbreviations: AKD, acute kidney disease; AKI, Acute kidney injury; SHR, subdistribution hazard ratio; CI, confidence interval; CKD, chronic kidney disease; ESRD, end-stage renal disease; † Adjusted with AKI stage, age, gender, body mass index, smoking, diabetes mellitus, hypertension, chronic kidney disease, coronary artery disease, mean arterial pressure, pre-operation creatinine, pre-operation hemoglobin, bypass time, shock at operation room, extensions for type A surgery.

Supplementary Table S4. The associated factors of AKI and AKD using the multivariable logistic regression analysis with backward elimination

Outcome / Variable	Adjusted OR (95% CI)	*P* value
AKI#		
Chronic kidney disease	2.20 (1.22 - 3.98)	0.009
Creatinine, mg/dL	1.88 (1.30 - 2.72)	<0.001
Hemoglobin, g/dL	1.14 (1.05 - 1.24)	0.003
Bypass time, min	1.005 (1.003 - 1.008)	<0.001
Aortic root replacement (Bentall operation)	0.41 (0.22 - 0.77)	0.006
AKD*		
AKI stage (Reference: Stage 0)		
1	1.91 (1.12 - 3.25)	0.018
2	4.14 (2.33 - 7.33)	<0.001
3	13.97 (8.15 - 23.92)	<0.001
Chronic kidney disease	1.73 (0.96 - 3.09)	0.066
Hemoglobin, g/dL	0.91 (0.82 - 1.01)	0.070

AKI, acute kidney injury; AKD, acute kidney disease; OR, odds ratio; CI, confidence interval;
# Variables included in backward selection logistic model with age, gender, body mass index, smoking, diabetes mellitus, hypertension, chronic kidney disease, coronary artery disease, mean arterial pressure, pre-operation creatinine, pre-operation hemoglobin, bypass time, shock at operation room, extensions for type A surgery;
* Variables included in backward selection logistic model with AKI stage, age, gender, body mass index, smoking, diabetes mellitus, hypertension, chronic kidney disease, coronary artery disease, mean arterial pressure, pre-operation creatinine, pre-operation hemoglobin, bypass time, shock at operation room, extensions for type A surgery.
